# Dietary oil composition differentially modulates intestinal endotoxin transport and postprandial endotoxemia

**DOI:** 10.1186/1743-7075-10-6

**Published:** 2013-01-10

**Authors:** Venkatesh Mani, James H Hollis, Nicholas K Gabler

**Affiliations:** 1 Department of Animal Science, Iowa State University, Ames, IA 50011, USA; 2 Interdepartmental Toxicology Graduate Program, Iowa State University, Ames, IA 50011, USA; 3 Department of Food Science and Human Nutrition, Iowa State University, Ames, IA, USA

**Keywords:** Dietary fat, Endotoxin, Intestine

## Abstract

**Background:**

Intestinal derived endotoxin and the subsequent endotoxemia can be considered major predisposing factors for diseases such as atherosclerosis, sepsis, obesity and diabetes. Dietary fat has been shown to increase postprandial endotoxemia. Therefore, the aim of this study was to assess the effects of different dietary oils on intestinal endotoxin transport and postprandial endotoxemia using swine as a model. We hypothesized that oils rich in saturated fatty acids (SFA) would augment, while oils rich in n-3 polyunsaturated fatty acids (PUFA) would attenuate intestinal endotoxin transport and circulating concentrations.

**Methods:**

Postprandial endotoxemia was measured in twenty four pigs following a porridge meal made with either water (Control), fish oil (FO), vegetable oil (VO) or coconut oil (CO). Blood was collected at 0, 1, 2, 3 and 5 hours postprandial and measured for endotoxin. Furthermore, ex vivo ileum endotoxin transport was assessed using modified Ussing chambers and intestines were treated with either no oil or 12.5% (v/v) VO, FO, cod liver oil (CLO), CO or olive oil (OO). Ex vivo mucosal to serosal endotoxin transport permeability (Papp) was then measured by the addition of fluorescent labeled-lipopolysaccharide.

**Results:**

Postprandial serum endotoxin concentrations were increased after a meal rich in saturated fatty acids and decreased with higher n-3 PUFA intake. Compared to the no oil control, fish oil and CLO which are rich in n-3 fatty acids reduced ex vivo endotoxin Papp by 50% (P < 0.05). Contrarily, saturated fatty acids increased the Papp by 60% (P = 0.008). Olive and vegetable oils did not alter intestinal endotoxin Papp.

**Conclusion:**

Overall, these results indicate that saturated and n-3 PUFA differentially regulate intestinal epithelial endotoxin transport. This may be associated with fatty acid regulation of intestinal membrane lipid raft mediated permeability.

## Background

The link between dietary fat and endogenous blood endotoxin has attracted increased medical and biomedical interest over the last few years. Furthermore, hyperphagia, increased adiposity and metabolic changes associated with high fat feeding can be recapitulated in mice chronically infused with LPS for four weeks [1]. It has been reported that the structure of fat consumed (emulsion vs. free oil) changes the extent of endotoxemia and that altering the composition, structure and quality of dietary fats could improve health [2]. In healthy humans, postprandial plasma endotoxin concentrations increase on average 18% after a high fat meal (approximately 380 kcal from fat, 42% of total energy) compared to the fasted state [3]. These authors concluded that increased postprandial LPS may contribute to the development of postprandial inflammation and disease. Ghanim et al. [4,5] also showed that in healthy adults, high fat, high carbohydrate meal (~900 kcal) increased postprandial plasma LPS concentrations by 70%. However, Laugerette et al. [6] recently reported that dietary oil composition differentially modulated murine inflammation and endotoxin transport. These authors also showed that fat composition, not quantity in the diet (22 vs. 3%) was critical in modulating plasma endotoxemia. Collectively, these data show that dietary fat intake and composition is able to modulate blood endotoxin and that this is associated with acute inflammation and the metabolic diseases of obesity and diabetes.

Both gram positive and gram negative bacteria are present in large quantities in the intestine. Interestingly, the total quantity of endotoxin, which is the gram negative bacterial outer cell wall component, in the intestine alone could be up to one gram [7]. Even very small quantities of endotoxin, pico-gram scale, in the systemic circulation has the potential to elicit an inflammatory response in humans and animals [8]. Endotoxin is also synonymously referred to as lipopolysaccharide (LPS), and both of these compounds are major immunogens that elicit an inflammatory response in numerous tissues and cell types via their recognition through pathogen-associated molecular patterns (PAMPs) and Toll like receptors in the innate immune system [9]. Endotoxin is thought to enter circulation by transport across the intestinal epithelium either via paracellular pathways through the openings of intestinal tight junctions between two epithelial cells or by a transcellular pathway [7]. Transcellular transport and the associated endocytosis of intestinal derived endotoxin may be facilitated by intracellular signaling processes mediated by the innate immune receptor complex CD 14/Toll like receptor 4 (TLR4)/MD-2, in association with the cell membrane micro domain lipid raft [10]. Furthermore, circulating endotoxin concentrations may also be augmented by transport coupled to dietary lipids and chylomicrons [11].

In recent years accumulating research has investigated the link between dietary fat and endogenous endotoxin in relation to metabolic inflammation [12,13]. Current evidence suggests that dietary fat augments circulating endotoxin concentrations and the resultant postprandial endotoxemia leads to low-grade systemic inflammation which has been implicated in the development of several metabolic diseases [1,3,14]. Intestinal derived endotoxin and the subsequent acute endotoxemia are considered major predisposing factors for inflammation associated diseases such as atherosclerosis, sepsis, obesity, type 2 diabetes and Alzheimer's [15-17]. However, the ability of different types of oil and fatty acids to facilitate uptake of intestinal endotoxin has been poorly characterized. Interestingly, saturated and n-3 polyunsaturated fatty acids (PUFA) have been shown to reciprocally modulate the LPS receptor, TLR4, and cell membrane lipid rafts [18]. This is postulated to be due to saturated fatty acids (SFA) such as lauric and myristic acid being part of the fatty acyl side chain composition of Lipid-A component of endotoxin and the ability of n-3 PUFA to reduce the potency of endotoxin when substituted in place of saturated fatty acids in lipid-A [19,20]. Thus, there is clear linkage between fatty acids (saturated, n-3 polyunsaturated, monounsaturated etc. …) and endotoxin signaling.

Therefore, the aim of this study was to assess the effects of various dietary fats on in vivo and ex vivo intestinal endotoxin transport and circulating concentrations using the pig as a biomedical model. We hypothesize that oils rich in saturated fatty acids (SFA) would augment, while the oils containing the n-3 PUFA (docosahexaenoic acid [DHA] and eicosapentaenoic acid [EPA]) would attenuate, intestinal endotoxin transport and postprandial endotoxemia.

## Methods

### Materials and animals

All the chemicals used for this study were purchased from Sigma-Aldrich (St. Louis, MO) unless otherwise stated. All animal use and procedures were approved by the Iowa State University Institutional Animal Care and Use Committee.

### Effect of dietary oil on postprandial serum endotoxin concentration

Twenty four pigs (49 ± 7 kg BW) were raised on a typical corn-soybean diet that met or exceeded their nutrient requirements [21] and randomly allocated to one of four treatments. The treatments consisted of 500 g ground corn-soybean meal dough (2,145 kcal ME) made up with either 1) 50 ml water (Control); 2) 50 ml fish oil (FO) (Spring Valley Inc., UT); 3) 50 ml vegetable oil (VO) (Hy-Vee Inc., IA) ; or 4) 50 ml coconut oil (CO) (Spectrum Naturals Inc., NY). After an overnight fast, six pigs were fed one of each porridge meal. Pigs voluntarily consumed the entire porridge meal with in ten minutes after the meal was offered. Blood was collected at 0, 1, 2, 3, and 5 hours postprandial by venipuncture using pyrogen free vaccutainer tubes and sterile needles. Proper precautionary measures were taken to prevent external endotoxin contamination of blood. Serum was separated by centrifuging at 2000 × g and 4°C. Serum was then stored in pyrogen free tubes at −80°C until further analysis.

Circulating serum endotoxin concentration was measured using the end point fluorescent assay using the recombinant factor C (rFC) system (Lonza^™^, Switzerland). Briefly, the serum samples were diluted 1000 times and 100 μl of the samples or standards were added to a 96 well plate and incubated at 37°C for 10 min. Thereafter, 100 μL of rFC enzyme, rFC assay buffer and rFC substrate were added at a ratio of 1:4:5 to the plate and an initial reading were taken followed by 1 h incubation at 37°C. The relative fluorescence unit (RFU) for each well was determined (excitation 380 nm and emission 440 nm). A positive control standard from the assay kit was used to ascertain the validity of the assay and the concentration of the endotoxin was interpolated from the standard curve constructed from the standards and corrected for sample dilution.

### Ex vivo intestinal integrity and endotoxin transport

Freshly isolated ileum segments from eleven pigs (21–28 days old) were placed in chilled Krebs-Henseleit buffer (consisting of, in mmol/L: 25 NaHCO_3_, 120 NaCl, 1 MgSO_4_, 6.3 KCl, 2 CaCl_2_, 0.32 NaH_2_PO_4_; pH 7.4) for transport to the laboratory while under constant aeration. Intestinal tissues were then stripped of their outer serosal layer and immediately mounted into modified Ussing chambers (Physiologic Instruments Inc., San Diego, CA and World Precision Instruments Inc. New Haven, CT). Each chamber and intestinal segment (0.71 cm^2^) was bathed on its mucosal and serosal sides with 5 ml of Krebs-Henseleit buffer and constantly gassed with 95% O_2_-5% CO_2_ mixture. Chambers were connected to a pair of dual channel current and voltage electrodes containing 3% noble agar bridges and filled with 3 M potassium chloride to measure electrophysiological parameters of the intestinal membranes or to measure the mucosal to serosal transport of endotoxin. Transepithelial resistance (TER) was not different across pigs, indicating no differences in paracellular permeability or leaky gut (data not shown).

To rule out any influence that bile acids may have on intestinal integrity, TER and macromolecule permeability was first tested on isolated ileum samples that were incubated with porcine bile acid (0, 3, 6 and 9 mg/ml) for thirty minutes. Thereafter, FITC-labeled dextran (FITC-Dextran, 4.4 kDa) mucosal to serosal transport was measured as described previously [22]. Briefly, the mucosal chambers were challenged with 2.2 mg/mL FITC-Dextran and chamber samples from both sides were collected every 10–15 min for eighty minutes. The relative fluorescence was then determined using a fluorescent plate reader (Bio-Tek, USA) with the excitation and emission wavelengths of 485 and 520 nm, respectively. Thereafter, an apparent permeability coefficient (Papp) was calculated for each treatment:

(1) $$\text{Papp}=\frac{\mathrm{dQ}}{\left({\mathrm{dt}\times A\times C}_{0}\right)}$$

Where: dQ/dt = transport rate (μg/min); C_0_ = initial concentration in the donor chamber (μg/ml); A = area of the membrane (cm^2^).

The effect of dietary fat on endotoxin transport was studied using ex vivo permeability of fluorescein isothiocyanate (FITC) labeled-LPS (Escherichia coli 055:B5) mounted into modified Ussing chambers. Briefly, segments of swine intestinal tissues were treated with either 12.5% (v/v) buffered saline control (CON), Fish Oil or cod liver oil (CLO) manufactured by Spring Valley Inc., UT) or vegetable oil, coconut oil or olive oil (OO) purchased form Hy-Vee Supermarkets Inc., IA). All oils were commercial retail available and then mixed with 20 mM sodium taurodeoxycholate (bile acid) for micelle formation to simulate the intestinal milieu. Each mucosal chamber was then challenged with 20 μg/mL FITC-LPS and chamber samples were collected every 10–15 min for eighty minutes. The relative fluorescence of each sample was then determined using a fluorescent plate reader (Bio-Tek, USA) with the excitation and emission wavelengths of 485 and 520 nm, respectively. The apparent permeability coefficient was then calculated similar to that described above for FITC-Dextran.

### Lipid rafts, dietary oil and ex vivo intestinal endotoxin transport

To examine the role of lipid rafts in intestinal endotoxin transport, ileum segments from 16 pigs (56 ± 4 days of age) were mounted in Ussing chambers as described above. Segments were pre-treated with or without 25 mM Methyl-β-cyclo dextrin (MβCD, a synthetic lipid raft modifier) for 30 min. Thereafter, the mucosal chamber was spiked with either saline-bile acid (CON) or Coconut oil-bile acid (12.5% v/v) and the FITC-LPS apparent permeability coefficient for each tissue was calculated.

### Fatty acid analysis

Fatty acid profiles of the dietary oils used to make the porridge were determined and analyzed by GC-MS [23,24]. One ml oil was mixed with 0.5 mL of 4:1 hexane and 125 μg/L heptadecanoic acid was added to each sample as an internal standard. FAME were analyzed by GC on a Hewlett-Packard model 6890 fitted with an Omegawax 320 (30-m × 0.32-mm i.id. 0.25 um) capillary column. Hydrogen was the carrier gas. The temperature program ranged from 80 to 250°C with a temperature rise of 5°C/min. The injector and detector temperatures were 250°C and 1 μL of sample was injected and run split. Fatty acids methyl esters were identified by their relative retention times on the column with respect to appropriate standards and heptadecanoic acid.

### Data analysis

Results are presented as means ± S.E.M and were analyzed with the Proc Mixed procedure of SAS (Cary, NC). In the model, repetition or day of Ussing chamber run was used as a random effect. Statistical significance of difference was analyzed by analysis of variance (ANOVA) followed by Tukey’s range test for pair wise comparison of all treatment means. Differences were considered significant at P ≤ 0.05 and a tendency at P ≤ 0.10.

## Results

### Dietary oil fatty acid profiles

The fatty acid composition of the oils used to make the porridge meal and/or in the ex vivo transport study are reported in Table 1. The coconut oil contained high concentrations of saturated fatty acids (89%), particularly lauric, myristic, palmitic acids. Olive oil contained a very high content of monounsaturated oleic acid and a moderate amount of palmitic acid, with a saturated fat content of 29%. Vegetable oil used in this study contained a high quantity (50%) of arachidonic acid (20:4n6), 32% oleic acid and 13% palmitic acid. The fish oil used consisted of 35% docosahexaenoic acid (DHA) and 19% eicosapentaenoic acid (EPA), while the cod liver oil contained 32% palmitic acid, 25% arachidonic acid, 8.6% EPA and 4.3% DHA. The n6:n3 ratio was highest in the olive oil > vegetable oil > cod liver oil > fish oil > coconut oil.

**Table 1:** Dietary oil fatty acid composition used to make the porridge (g/100 g FA)

	**Oil Source**				
**Compound**	**Coconut oil**^**1**^	**Fish oil**^**2**^	**Olive oil**^**3**^	**Vegetable oil**^**4**^	**Cod liver oil**^**5**^
8:0	8.22	0.00	0.01	0.00	0.00
10:0	6.98	0.00	0.00	0.00	0.00
12:0	36.51	0.00	0.05	0.03	0.00
14:0	21.00	6.87	0.32	0.09	4.62
16:0	11.87	14.78	25.74	12.84	32.24
16:1	0.02	8.40	1.67	0.10	4.19
18:0	3.61	2.99	2.60	5.67	4.02
18:1	8.74	8.51	55.69	25.89	12.65
18:2 n6	1.93	0.92	12.66	47.19	25.28
18:3 n3	0.00	0.40	0.54	6.92	4.05
20:5 n3	0.00	19.19	0.00	0.00	8.68
22:6 n3	0.00	34.57	0.00	0.00	4.27
Other	1.11	3.38	0.71	1.26	0.00
Saturated	88.96	24.63	29.20	19.70	40.88
n3	0.00	54.17	0.54	6.92	17.00
n6	1.93	1.99	12.66	47.19	25.28
n6:n3	-	0.04	23.37	6.82	1.49

^1^Source coconut oil was (Spectrum Naturals, NY).

^2^Source fish oil was (Spring Valley, UT).

^3^Source olive oil was (Hy-Vee, IA).

^4^Source vegetable oil was (Hy-Vee, IA).

^5^Source cod liver oil was (Spring Valley, UT).

### Effect of dietary oil on postprandial serum endotoxin concentration

**Figure 1:**
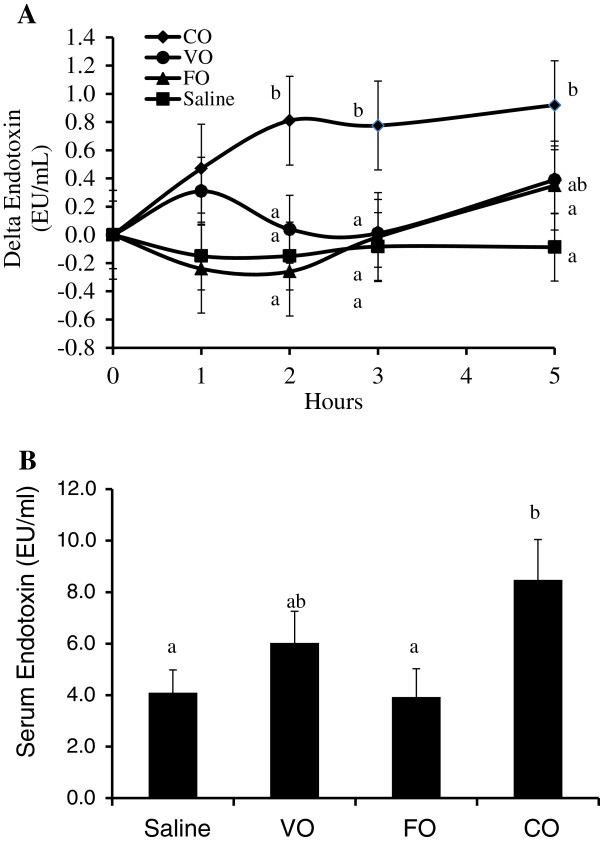
**Dietary oil alters postprandial serum endotoxin concentrations in pigs fed a single dietary oil-based meal. A) **Delta change in serum endotoxin concentrations. **B) **Mean postprandial serum endotoxin concentration. Different letters (**a**,**b**) represent significant difference at P < 0.05. Treatments are a porridge meal made with either no oil (saline), fish oil (FO), vegetable oil (VO) and coconut oil (CO). n = 6 pigs/treatment. Data are means ± S.E.M.

To assess the effect of dietary lipids on postprandial serum endotoxin concentrations, pigs received a porridge meal containing either 50 mL of saline, CO, VO or FO. The endotoxin concentration of the various oils used did not differ (data not shown). Change in postprandial serum endotoxin concentration due to different meal treatments are presented in Figure 1A. The overall postprandial serum endotoxin concentrations were significantly lower in the meals constituting saline or FO, with the mean overall serum endotoxin concentration increasing two-fold over the saturated coconut oil meal treatment (P < 0.05, Figure 1B). However, meals made up with VO were not different from the saline, CO or FO treatments (P < 0.05). Interestingly, the CO meal significantly elevated serum endotoxin concentrations after 2 hours versus the saline and FO, and these remained elevated at 3 and 5 hour postprandial (P < 0.05, Figure 1A).

### Effect of exogenous porcine bile acid on ex vivo intestinal integrity

**Figure 2:**
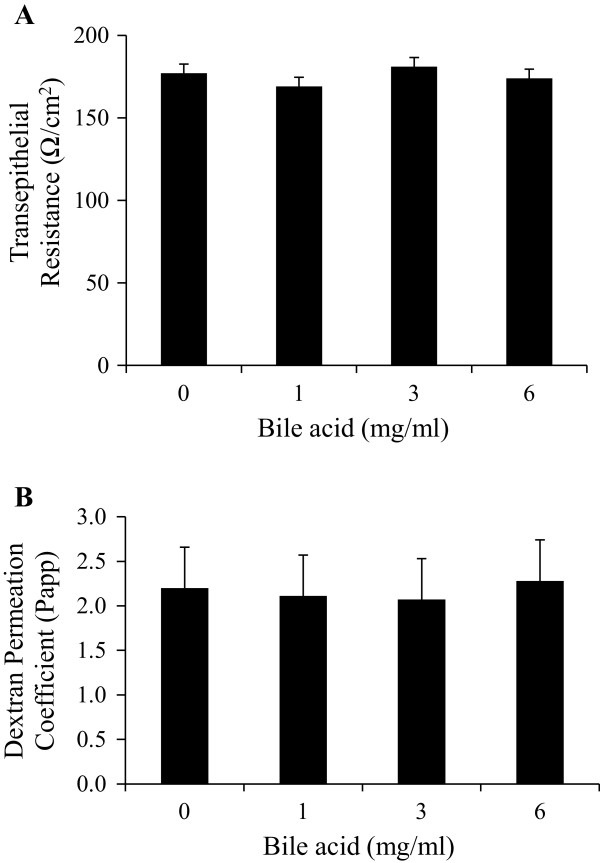
**The effect of increasing porcine bile acid concentration on ex vivo intestinal integrity and permeability. A) **Transepithelial resistance (TER) and **B) **FITC-Dextran transport (4.4 kDa). Freshly isolated ileum samples were mounted into modified Ussing chambers and incubated with the indicated concentration of bile acid for 30 minutes and then FITC-Dextran was added to mucosal side. Permeation coefficient was calculated by taking samples from chambers every 10–15 minutes and measuring the amount of fluorescence. Different letters represent significant difference at P < 0.05. n = 11 pigs. Data are means ± S.E.M.

Bile acids have been shown to increase the intestinal permeability in cultured Caco-2 cell lines [25]. To rule out the effect that exogenous bile acid may reduce intestinal integrity, freshly isolated pig ileum segments were used to measure TER (Figure 2A) and FITC-Dextran permeability (Figure 2B). As these segments were exposed to increasing concentrations of porcine bile ex vivo, no differences in intestinal integrity were observed (P > 0.10, Figure 2). This might be due to the tolerance of intestinal tissues towards bile acid because of previous exposure in vivo contrary to cell cultures where the cells are not exposed to the bile acids previously.

### Effect of dietary oil on ex vivo intestinal endotoxin transport

**Figure 3:**
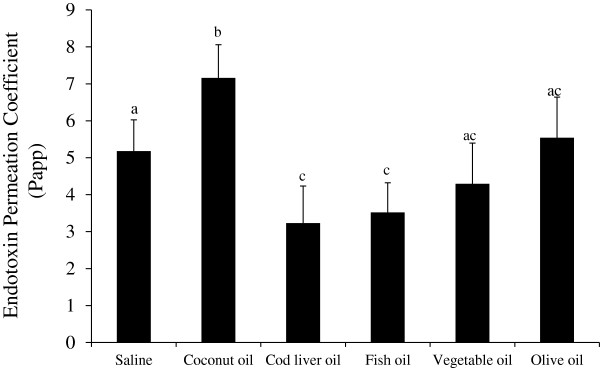
**Ex vivo endotoxin transport in pig ileum tissue exposed to different dietary oil treatments. **Freshly isolated ileum samples were mounted into modified Ussing chambers and mixed with the indicated oils and 20 mM bile acid for 120 minutes and FITC-LPS transport was measured. Different letters represent significant difference at P < 0.05. n = 11 per treatment. Data are means ± S.E.M.

The ex vivo mucosal to serosal ileum endotoxin transport was assessed using modified Ussing chambers and FITC-LPS permeability assay (Figure 3). Compared to the saline no oil control treatment, the endotoxin Papp was significantly lower in both the FO and CLO treatments (P < 0.05). As hypothesized, the higher saturated fat content of coconut oil significantly increased the endotoxin Papp compared to the saline, FO and CLO (P < 0.05). However, mucosal treatment with VO and OO did not differ from the saline or n-3 treatments (P > 0.05), but still attenuated endotoxin Papp versus the coconut oil treatment (P < 0.05, Figure 3). Transepithelial resistance was not different due to ex vivo oil treatment (data not shown).

### Effect of lipid raft modification on saturated fat induced endotoxin transport

**Figure 4:**
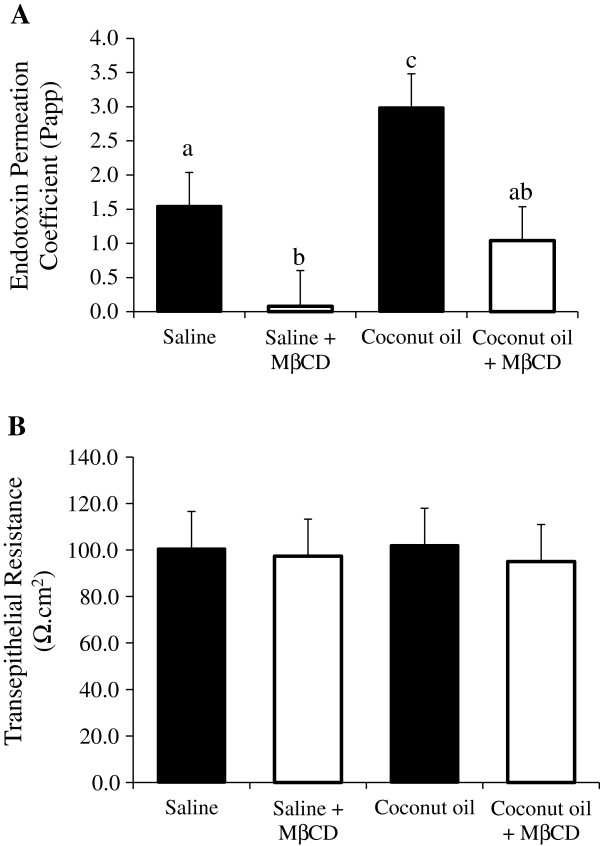
**Lipid raft modifier methyl beta cyclodextrin (MβCD) decreases ex vivo endotoxin transport. A) **Endotoxin transport and **B)** transepithelial resistance was measured using Ussing chambers in ileum tissues treated with either control (water), MβCD, coconut oil, or coconut oil plus MβCD. Tissue (n = 7 /trt) were pretreated with these treatments for 30 min before FITC-LPS transport was assessed. Different letters represent significant difference at P < 0.05. Data are means ± S.E.M.

To test the hypothesis that destabilization of intestinal lipid rafts would decrease saturated fat induced endotoxin permeability, ileum samples were pretreated with the lipid raft modifier methyl-β-cyclodextrin (MβCD) and coconut oil ex vivo. FITC-LPS endotoxin transport permeability was then measured (Figure 4A). As expected, the CO treatment significantly augmented the ileum endotoxin Papp compared to the saline control (P < 0.05). However, the endotoxin Papp was significantly reduced with the MβCD treatment compared to the saline control (1.54 vs. 0.07, P = 0.04). In the presence of MβCD and CO, the ileum Papp was attenuated three fold from the CO alone treatment (P < 0.05). Importantly, ileum integrity and permeability as measured by transepithelial resistance was not altered by either short term coconut oil, MβCD, or the combination compared to the saline control (P = 0.98, Figure 4B).

## Discussion

In Western diets, vegetable, canola and palm oils are common components of the diet and to a lesser extent, long chain n-3 PUFA (DHA and EPA) oils from algal or marine sources [26,27]. In recent years, the development of obesity, inflammation, atherosclerosis and other metabolic diseases has been linked to low grade endotoxemia associated with high dietary fat and energy intake [3,14,28-30]. However, these studies and others have raised questions on whether this diet induced endotoxemia reflects changes in energy and fat content of the diet, intestinal permeability or diet induced changes in gut microbiota. In the current study, we used ex vivo and in vivo methods to examine intestinal permeability to endotoxin as it relates to dietary oil composition. All pigs were clinically healthy and raised on typical commercial swine corn-soybean diets. We observed no differences in intestinal integrity due to our ex vivo treatments. Even though some cell culture experiments have been shown to indicate bile acids affecting the intestinal permeability, we didn’t observe any negative impacts from bile acids on intestinal integrity and permeability. Further, Lang et al. reported that bile alone may not be sufficient to induce barrier function in the Ex-vivo experiments [31]. Importantly, we only examined the acute actions of a meal or oil bolus treatment and did not conduct a prolonged feeding trial in an attempt to change the pig microbiota populations or the fatty acid profiles of tissues due to diet.

We hypothesized that dietary intake of oils rich in DHA and EPA would attenuate intestinal endotoxin transport and postprandial circulating endotoxin. We found that dietary cod liver and fish oils attenuated serum endotoxin concentrations compared to the coconut oil and the endotoxin levels in these pigs were similar to the control group (Figure 1). To the best of our knowledge, there are no other studies that have shown this effect of DHA and EPA on endotoxin transport and blood endotoxemia.

Interestingly, only one paper has examined the effects of dietary oil composition on endotoxin uptake and related inflammation [6]. However, contrary to our results, the report by Laugerette et al. [6] states that rape seed (canola) and sunflower oil, with its high unsaturated fatty acid content, augmented plasma endotoxemia by 50-75%. Cani et al. [1], also observed a similar increase in endotoxemia in mice orally administered corn oil with or without LPS compared to water alone. However, we observed no change in serum postprandial endotoxin concentration or intestinal endotoxin transport due to dietary vegetable oil compared to the saline control (Figures 1 and 3). This discrepancy of these parameters between the studies might be partially explained by the use of different animal models and also the nature of the experimental design. Whereas Laugerette et al. [6] performed a chronic feeding study using mice for eight weeks using oils with different fatty acid composition; we used an acute meal bolus model with swine to examine endotoxin permeability. Therefore, the mice fatty acid profiles would have mimicked their diets and this tissue enrichment may also modify endotoxin permeability, signaling and acute inflammation. Further work is needed to explain the molecular aspects of these differences between the two studies with regard to acute versus chronic dietary fat signaling in the gastrointestinal tract. Additionally, we observed a significant increase in postprandial endotoxemia after a porridge meal mixed with coconut oil (Figure 1). Again, this contradicts data presented by Laugerette et al. [6] in which palm oil, high in saturated fatty acids, had no effect on plasma endotoxin concentrations in mice. However, these authors did report an increase in plasma LPS binding protein and argued that this protein is a better marker of endotoxemia due to the short half-life of circulating endotoxin. One issue is that LPS binding protein can be up regulated by inflammation and acute stress as well as both gram positive and negative infections. As the magnitude of LBP response goes down with multiple episodes of infection [32], this could be a result of the agonistic effects of saturated fatty acid on pro-inflammatory signaling and not circulating endotoxin.

Gram negative bacteria, particularly those found in the distal ileum and colon, might be one of the major sources for circulating endotoxin [33]. It has been estimated that a single cell of *Escherichia coli* contains approximately 10^6^ Lipid A or endotoxin molecules and a typical human intestinal tract could harbor approximately one gram of endotoxin [33-35]. Interestingly, the bacterial population in the intestine is not static. Multiple studies have shown that bacterial composition shifts to either gram positive majority or gram negative majority based on the composition of the diet consumed [30,36,37]. A majority of these studies show that consuming high saturated fat diet for a longer period results in higher gram negative bacterial populations and high fiber diets results in gram positive bacterial populations [30,38]. Laugerette et al. [6], reaffirmed this and showed that fatty acid composition of different dietary oils can alter intestinal microbiota populations. Moreover, these authors demonstrated that feeding a diet high in palm oil which is rich in SFAs significantly increased the gram negative bacteria *Escherichia coli* groups, which can be significant source of endotoxin in the cecal content of mice compared to milk fat, rape seed and sunflower oil fed diets.

During intestinal stress, ischemia, inflammation and diseases, paracellular transport occurs through the tight junction, as known as “leaky gut” [39]. Alternatively, transcellular or intracellular transport can occur, particularly in healthy individuals [40]. Transcellular endotoxin transported across a cell membrane has been shown to occur via TLR4 and soluble GPI anchored receptor CD14 in a lipid raft mediated mechanism [41,42]. Additionally, chylomicron associated LPS transport has also been suggested to play a key role in intestinal LPS transport from the intestinal epithelial cell [11,43,44]. Importantly, we observed no decrease in intestinal integrity which might enhance paracellular permeability as assessed by transepithelial resistance or FITC-dextran permeability due to treatment or short term raft destabilization (Figure 4B). These data suggest that under healthy intestinal epithelial conditions, endotoxin is most likely transported via lipid raft mediated endocytosis.

The signaling and transport process for endotoxin is initiated in specialized membrane micro domains called lipid rafts [42]. Lipid rafts are membrane regions rich in cholesterol, glycolipids, sphingolipids and saturated fatty acids, which result in a ‘rigid’ membrane structure compared to the adjacent ‘fluid’ regions [45]. In immune cells, endotoxin triggers the recruitment of TLR4 into the lipid raft, where it interacts with CD14 and other associated proteins such as MD-2 resulting in an inflammatory signaling cascade [46,47]. Thus, the two major consequences of preventing endotoxin recognition by dissociating the lipid raft to attenuate TLR4 recruitment include reduced inflammatory signaling and attenuated endotoxin transport. We observed that if intestinal lipid rafts are dissociated ex vivo with MβCD, then endotoxin permeability is attenuated in the ileum (Figure 4). Interestingly, saturated fat induced endotoxin permeability is also significantly reduced. Stimulation of TLR4 receptor has been shown to result in the endotoxin transport across the intestinal epithelial cells [40]. TLR4 is not only implicated in the transcellular transport of LPS but also for live bacteria [48]. Since saturated fatty acids and n-3 PUFA can reciprocally modulate TLR4 signaling [49], the fatty acid composition of oil in the diet has the potential to increase or decrease endotoxin transport. Altogether, these data suggest that apical endotoxin transport in the intestines is arguably raft mediated in healthy individuals.

In vitro experiments show clearly that n-3 PUFA disrupt TLR4 signalling and the activation of NFκB by LPS in a murine monocytic cell line [50]. Moreover, DHA modulates TLR4 signaling in vitro in RAW 264.7 macrophages and 293 T cells [49], human monocytes and dendritic cells [51] and adipose tissue. We have previously shown in pigs that dietary EPA and DHA are effective means of influencing the inflammatory status and pathways influenced by TLR4 signaling induced by LPS [52] and in altering intestinal function [24,53]. Therefore, one could postulate that antagonizing TLR4 recruitment to lipid rafts and it’s signaling by DHA and EPA, or stimulating these processes with saturated fatty acids, would alter endotoxin transport and circulating postprandial endotoxin.

Another mechanism through which endotoxin can enter the circulation is through micelles. Since the endotoxin side chains are made up of fatty acids, endotoxins can be incorporated into the micelles and transported into the intestinal epithelial cell [54]. In intestinal epithelial cells, chylomicrons transport the absorbed lipids into various parts of the body. High fat administration has been shown to proportionately increase the endotoxin content of the chylomicron indicating that high fat consumption indeed enhances higher endotoxin transport into the intestinal epithelial cell and incorporation into chylomicron [11,28]. Furthermore, even though the mechanism is not clear, high intake of fat has been shown to cause internalization of tight junction proteins and increase in the paracellular permeability to macro molecules including endotoxin [30]. Even though, this mode of endotoxin transport cannot be ruled out, we speculate that the rate of incorporation of fatty acids into micelles would not vary due to oil composition. Therefore, we propose that the difference in intestinal endotoxin transport we observed is primarily transcellular transport that involves lipid rafts and receptor mediated endocytosis [42].

In conclusion, these data suggest that dietary oils can differentially alter intestinal endotoxin transport. Oils rich in DHA and EPA attenuate endotoxin transport, while oils high in saturated fatty acids augment endotoxin transport. Furthermore, intestinal endotoxin transport in healthy subjects may be regulated through a lipid raft mediated mechanism. Saturated fatty acids may be stabilizing the lipid rafts allowing for greater endotoxin transport.

## Abbreviations

CON: Control; VO: Vegetable oil; CLO: Cod liver oil; CO: Coconut oil; OO: Olive oil; FITC: fluorescein isothiocyanate; TLR4: Toll like receptor 4; TER: Transepithelial resistance; LPS: Lipopolysaccharide; FA: Fatty acids; PAMPs: Pattern associated molecular patterns; PUFA: Polyunsaturated fatty acids; SFA: Saturated fatty acids; Papp: Apparent permeability co-efficient; FAME: Fatty acid methyl esters; MβCD: Methyl-beta-cyclo dextrin; rFC: Recombinant factor C; RFU: Relative fluorescent unit; DHA: Docosahexaenoic acid; EPA: Eicosapentaenoic acid; IEC: Intestinal epithelial cell.

## Competing interests

The authors declare that they have no competing interests.

## Authors’ contributions

VM, JH and NKG designed and conducted research presented. NKG was the principle investigator and both NKG and JH obtained funding for this work. VM was the graduate student supervised by NKG whom conducted most of the animal and laboratory work and wrote the manuscript. JH and NKG supervised and revised the manuscript. All authors read and approved the final manuscript.
